# Tumeur unguéale rare: le fibrome molluscum ou fibrome mou à propos d'un cas

**DOI:** 10.11604/pamj.2015.20.289.6592

**Published:** 2015-03-25

**Authors:** Soufiane Guelzim, Mustapha Mahfoud

**Affiliations:** 1 Service de Chirurgie Orthopédique et Traumatologie, CHU Ibn Sina, Rabat, Maroc

**Keywords:** Tumeur, ongle, fibrome mou, Tumor, nail, soft fibroma

## Image en medicine

Le fibrome mou ou fibrome molluscum est une tumeur bénigne se développant à partir de tissu conjonctif. Il peut toucher tous les organes. Nous rapportons des images cliniques, paracliniques et opératoires d'un fibrome mou de localisation unguéale inhabituelle. Il s'agit d'une patiente de 45 ans, qui s'est présentée à la consultation pour un nodule mou, pédiculé, de couleur chair, indolore, localisé sur l'ongle du 4ème doigt de la main droite. Elle ne présentait aucune douleur, pas de déficit sensitif, pas d'inflammation locale et il n'y avait pas d'autres localisations. La radiographie était normale. A cause d'une gêne esthétique rapportée par la patiente, Une exérèse chirurgicale avec ablation de la tablette unguéale sous jacente été réalisée, sous anesthésie locale, avec étude anatomopathologique qui a confirmé le diagnostic. À 6 mois, la repousse unguéale était bonne et sans récidive.

**Figure 1:**
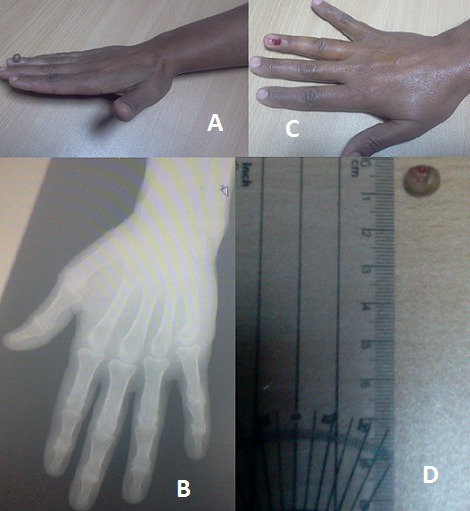
(A): image clinique: nodule mou pédiculé, couleur brune, unguéal au niveau 4ème doigt de la main droite; (B): radiographie main droite: normale; (C): image clinique après résection chirurgicale; (D): image de la tumeur réséquée envoyée pour étude anatomopathologique

